# Research Progress on Preparation Technology, Structure Optimization and Properties of 3D-Printed Porous Ceramics

**DOI:** 10.3390/ma19122674

**Published:** 2026-06-22

**Authors:** Qintao Shen, Peng Wang, Chao Ding, Chunan Song, Yapeng Ning, Renquan Ji, Jiatao Du, Viboon Saetang, Xiaojing Li, Junyi Pan, Yaxuan Wei, Jiying Wang, Xin Yang, Huan Qi

**Affiliations:** 1 Zhejiang Key Laboratory of Aerospace Metallic Materials, Hangzhou City University, Hangzhou 310015, China; sqt@hzcu.edu.cn (Q.S.); wangp@hzcu.edu.cn (P.W.); dingchao@hzcu.edu.cn (C.D.); ningyp@hzcu.edu.cn (Y.N.); jirq@hzcu.edu.cn (R.J.); 22525264@zju.edu.cn (J.D.); 2 Zhejiang-Thailand International Joint Laboratory on New Materials Digital Design and Processing Technology, Hangzhou City University, Hangzhou 310015, China; viboon.tan@kmutt.ac.th; 3 School of Engineering, Hangzhou City University, Hangzhou 310015, China; 4 School of Mechanical Engineering, Zhejiang University, Hangzhou 310030, China; 5 School of Materials Science and Engineering, Zhejiang University, Hangzhou 310030, China; 13227788157@163.com; 6 Department of Production Engineering, Faculty of Engineering, King Mongkut’s University of Technology Thonburi, Bangkok 10140, Thailand; 7 Zhejiang Metallurgical Research Institute Co., Ltd., Hangzhou 310011, China; lixiaojing@zmri.com.cn (X.L.); panjunyi@zmri.com.cn (J.P.); weiyx19@tsinghua.org.cn (Y.W.); wangjiying@hzsteel.com (J.W.)

**Keywords:** 3D printing, porous ceramics, preparation technology, structure optimization, performance regulation

## Abstract

Porous ceramics have garnered widespread attention in high-temperature insulation, aerospace, and other fields due to their excellent thermal stability, low density, and superior thermal insulation performance. However, traditional preparation technologies suffer from limitations such as poor pore structure controllability, unstable mechanical properties, and long production cycles. In recent years, 3D printing (additive manufacturing) technology has emerged as a disruptive approach to address these challenges, enabling precise fabrication of porous ceramics with complex structures and tailored properties. This review comprehensively summarizes the research progress on 3D-printed porous ceramics, focusing on preparation technologies, structure optimization, and performance regulation. First, the principles and drawbacks of traditional preparation methods are analyzed. Then, four mainstream 3D printing technologies (Binder Jetting, Material Extrusion, Vat Photopolymerization, and Material Jetting) for porous ceramics are elaborated on in terms of forming mechanisms, process characteristics, typical cases, and performance advantages/disadvantages. Additionally, the structure–property optimization strategies, including the design of Triply Periodic Minimal Surface structures and the application of computational modeling and simulation, are discussed to achieve the balance between thermal insulation and mechanical properties. Finally, current challenges and future development trends of 3D-printed porous ceramics are prospected. This review provides a systematic reference for the rational selection of preparation technologies, structural design, and performance optimization of porous ceramics, promoting their engineering applications in high-value fields.

## 1. Introduction

3D printing, also known as additive manufacturing (AM), has emerged as a revolutionary technology that is reshaping the research and application landscape of new materials [1,2,3]. Unlike traditional subtractive manufacturing methods that rely on cutting, grinding, or molding, 3D printing builds objects layer by layer from digital models, which not only reduces material waste but also enables the fabrication of complex structures that are difficult or impossible to achieve with conventional techniques [4,5,6,7]. In the field of new materials, 3D printing plays an increasingly important role, especially in promoting the development and application of metals, ceramics, composites, and other advanced materials [8,9,10,11,12,13], driving innovations in industries such as aerospace, automotive, medical, and energy [14,15,16].

In the metal materials field, 3D printing has broken traditional processing limits to become a key technology for high-performance metal components [17,18,19], with Laser Powder Bed Fusion (LPBF) and Electron Beam Melting (EBM) as the most mature and widely used industrial technologies [20,21]. LPBF uses a high-energy laser to selectively melt metal powder layer by layer, featuring high forming precision, good surface quality and broad material adaptability, ideal for high-value complex parts, boosting material utilization to over 90% and optimizing component mechanical properties [22,23]. By contrast, EBM melts powder with a high-energy electron beam in a vacuum, having higher energy density, lower residual stress and avoiding material oxidation, making it suitable for large, thick-walled components [24,25,26]. The vacuum environment of EBM effectively avoids the oxidation of metal materials during the melting process, ensuring the purity and performance of the prepared components [27,28]. Both technologies drive the development of hard-to-machine metals and customized metal matrix composites [29].

In addition to metal materials, 3D printing also shows unique advantages in the application of ceramic materials. Traditional ceramic processing is limited by the brittleness and hardenability of ceramics, making it difficult to prepare complex-shaped ceramic components. 3D printing technologies such as Stereolithography (SLA) and Binder Jetting (BJ) have solved this problem effectively. For example, SLA uses an ultraviolet laser to cure ceramic slurry layer by layer, which can prepare ceramic components with high precision and complex structure, such as ceramic molds, biological ceramic scaffolds, and electronic ceramic parts. BJ uses an adhesive to bond ceramic powder layer by layer, and then obtains dense ceramic components through sintering, which is suitable for mass production of ceramic parts with low cost. 3D printing has not only expanded the application scope of ceramic materials but also promoted the development of new ceramic materials such as gradient ceramic materials and porous ceramic materials [30].

**Figure 1:**
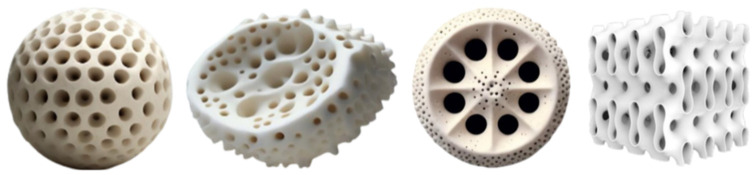
Porous ceramics.

High-temperature industrial processes account for approximately 40% of global energy consumption [31,32,33]. Reducing heat loss in high-temperature environments and improving energy utilization efficiency are crucial issues in the current industrial field [34,35]. The adoption of high-efficiency thermal insulation materials to minimize unnecessary heat transfer [36,37] is one of the key strategies for energy conservation. Among various materials, ceramic materials are widely used as refractory and thermal insulation materials due to their properties such as non-combustibility, high-temperature resistance, and corrosion resistance [38,39]. Compared with conventional dense ceramic materials, porous ceramics (as shown in Figure 1) possess a large number of interconnected or closed pores internally. This endows them with low density, high specific surface area, and high toughness, along with excellent thermal shock resistance, thermal insulation capability, and temperature stability. Among these, the structural attributes of porous ceramics, particularly porosity, pore size, and pore wall thickness, are the key factors determining their performance [40]. Different pore structures enable the application of porous ceramics in various functional directions. For instance, closed pore structures help hinder the flow of fluids inside porous ceramics, and when combined with high porosity, they can achieve excellent thermal insulation performance [41,42]. The superior properties of porous ceramics have led to their wide application in industries such as new energy vehicles, aerospace, construction, nuclear engineering, petrochemicals, and metallurgy [14,43].

Porous ceramics were initially fabricated by molding, drying, and sintering high-temperature ceramic powders [44,45]. However, this method often fails to precisely control pore size and porosity, limiting the practicality of the materials. Subsequently, the emergence of emerging technologies such as the sacrificial template method, direct foaming method, and 3D printing has greatly enhanced the flexibility and diversity of porous ceramic production, enabling the precise regulation of pore size and porosity [46,47,48]. In particular, 3D printing technology can directly produce porous ceramic products, simplifying the production process, significantly improving production efficiency, and overcoming many limitations of traditional die molding.

To systematically elaborate on the aforementioned research gaps and prospects, this review first summarizes the principles, characteristics, advantages, and inherent limitations of traditional porous ceramic preparation technologies, including partial sintering, freeze drying, direct foaming, and the sacrificial template method. Subsequently, this review focuses on four mainstream 3D printing technologies suitable for porous ceramic fabrication—Binder Jetting, Material Extrusion, Vat Photopolymerization, and Material Jetting (MJ)—detailing their forming mechanisms, process parameters, typical applications, and performance trade-offs. Then, it further explores the structure–property optimization strategies of 3D-printed porous ceramics, emphasizing the role of computational modeling and simulation in tailoring pore structures to balance thermal insulation and mechanical properties. Finally, it concludes the key findings of this review and proposes future research directions, aiming to provide a comprehensive reference for the advancement of high-performance 3D-printed porous ceramics in industrial applications.

## 2. Traditional Preparation Technologies of Porous Ceramics

The preparation of porous ceramics requires precise control of their pore structure parameters to meet specific application requirements [49]. Traditional preparation technologies include partial sintering, freeze drying, direct foaming, and the sacrificial template method, all of which can regulate the porosity and pore morphology of porous ceramics.

### 2.1. Partial Sintering

**Figure 2:**
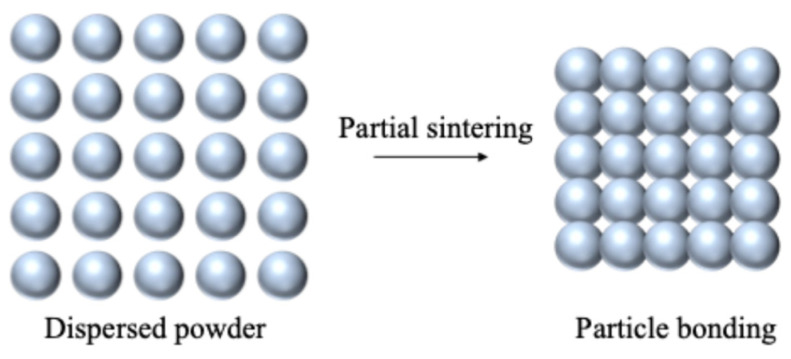
Schematic diagram of partial sintering [55].

Partial sintering [50,51,52] typically relies on the initial powder particle size, sintering temperature, and holding time to achieve limited sintering of powder particles only at contact points. This retains a certain amount of pores inside the material, forming a uniform porous structure. Its basic working principle is illustrated in Figure 2. Although the partial sintering method has a simple structure and does not require additional pore-forming agents, the formed porous structure is relatively random and cannot be precisely controlled. Additionally, due to incomplete particle bonding, the mechanical properties of the formed product are poor [53,54].

### 2.2. Freeze Drying

**Figure 3:**
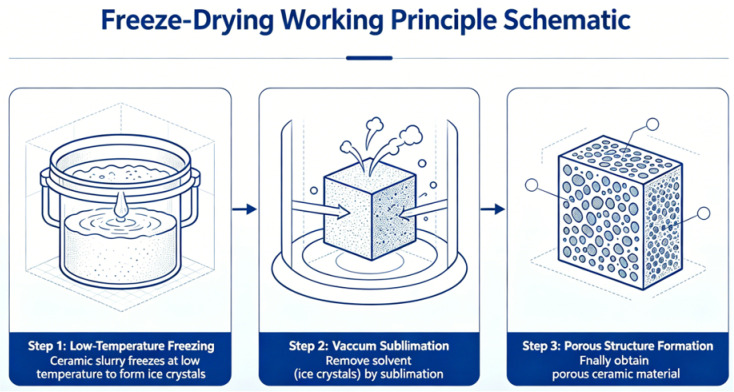
Schematic diagram of freeze drying.

The working principle of freeze drying is shown in Figure 3. Ceramic slurry is frozen to form ice crystals from the solvent at low temperatures, and then the solvent is removed by sublimation to form a porous structure [56,57,58]. Compared with partial sintering, freeze drying has relatively mature pore control during the forming process [53], but it has significant drawbacks such as a long preparation cycle, unidirectional pore structure, and low mechanical strength [59,60].

### 2.3. Direct Foaming

**Figure 4:**
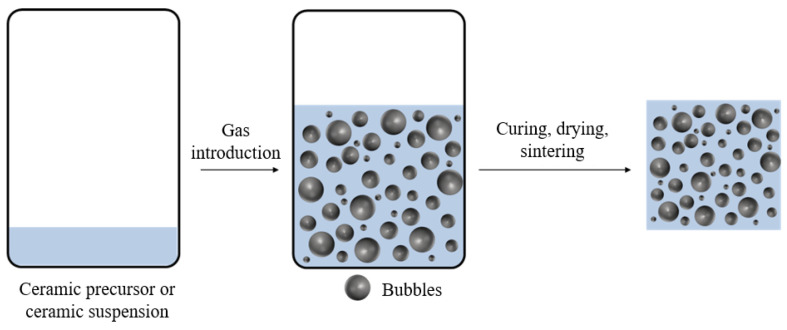
Schematic diagram of the direct foaming method [55].

As shown in Figure 4, direct foaming [61,62,63] is a porous ceramic forming method that introduces gas (e.g., through mechanical stirring, chemical reactions, or foaming agents) into ceramic slurry to form stable foams, and retains the pore structure during subsequent curing and sintering. The direct foaming method for forming porous ceramics has a simple process and is suitable for large-scale production. However, the stability of the foam is greatly affected by sintering temperature, slurry viscosity, and foaming agent performance, which easily leads to problems such as pore structure collapse or unevenness, resulting in poor final forming performance [42,64].

### 2.4. Sacrificial Template Method

**Figure 5:**
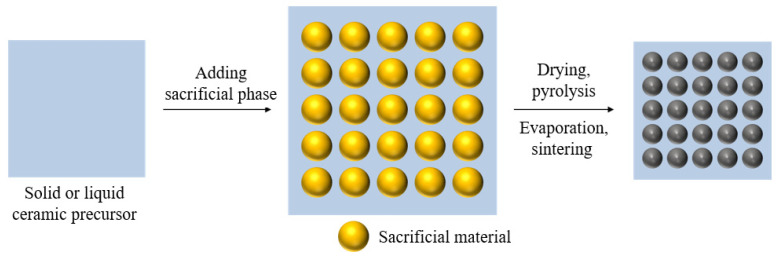
Schematic diagram of the sacrificial template method [55].

The sacrificial template method [46,65] is a mature porous ceramic forming method. It uses removable templates made of organic foams, polymer microspheres, and biological materials as the ceramic skeleton. The template is then removed by pyrolysis, dissolution, or gasification to form the desired porous ceramic structure. Its basic principle is shown in Figure 5. The main feature of this technology is that the pore structure can be further controlled according to the quantity, size, and geometric shape of the sacrificial template, forming porous ceramics with gradient pores [42]. However, in this process, in addition to the complex process, the decomposition of the sacrificial template may result in uncontrollable residual impurities [66,67], and the performance of the formed porous ceramics is greatly affected by the template material.

In summary, traditional porous ceramic preparation methods, including partial sintering, freeze drying, direct foaming and the sacrificial template method, all inevitably exhibit inherent and insurmountable limitations in practical applications. Partial sintering yields randomly distributed pore structures with unmanageable pore size and porosity; freeze drying results in unidirectional pore morphologies alongside low mechanical strength and an overly long preparation cycle; direct foaming is prone to pore structure collapse and inhomogeneity due to the sensitivity of foam stability to process parameters; and the sacrificial template method involves complex fabrication procedures, with the decomposition of templates leading to uncontrollable residual impurities. Collectively, these drawbacks give rise to poor designability and uniformity of the pore structure, unstable thermal insulation and mechanical properties of the fabricated ceramics, as well as inefficiencies in production that manifest as long preparation cycles and high manufacturing costs. Such deficiencies severely restrict the application of traditionally prepared porous ceramics in high-precision, high-performance and high-value scenarios such as aerospace high-temperature insulation and advanced structural–functional integrated components. In sharp contrast, the rapid rise of 3D printing (additive manufacturing) technology has fundamentally addressed these pain points of traditional preparation methods and opened up new possibilities for the fabrication of porous ceramics. Relying on computer-aided design (CAD) and layer-by-layer material deposition, 3D printing enables precise and customizable regulation of key pore structure parameters including pore size, porosity, morphology and interconnectivity, and realizes the one-step fabrication of complex and novel pore structures (e.g., Triply Periodic Minimal Surface structures and hierarchical porous structures) that are difficult or even impossible to achieve with traditional techniques. More importantly, 3D printing technology can synergistically optimize the thermal insulation and mechanical properties of porous ceramics according to actual application requirements by adjusting printing process parameters and structural design, breaking the performance trade-off dilemma faced by traditional methods. In addition, it eliminates the need for complex die molding, simplifies the production process, significantly shortens the manufacturing cycle, and balances high forming performance with the potential for scaled production. Ultimately, 3D printing technology has made the design of complex pore structures, high forming performance and mass production of high-precision, multi-scale and multi-functional porous ceramics a practical reality, laying a solid foundation for their engineering application in cutting-edge fields.

## 3. 3D Printing Preparation Technologies of Porous Ceramics

In practical applications, porous ceramics often need to have certain mechanical properties. Since pore structure characteristics (including high porosity, small pore size, uniform pore distribution, and closed pore morphology) enhance thermal insulation performance while reducing overall strength, balancing “high thermal insulation performance” and “reliable mechanical performance” has become a key challenge.

3D printing technology, also known as additive manufacturing technology, has been listed by the McKinsey Global Institute as one of the disruptive technologies that will shape future economic development [68], and has enormous potential in the preparation of porous ceramics. 3D printing technology directly models complex porous structures through Computer Aided Design (CAD), then physically forms them by layer-by-layer stacking of materials, combined with post-processing to meet specific process requirements, and finally forms porous ceramics with functional structures [69]. By precisely controlling the pore shape, size distribution, and interconnectivity of the formed porous structure, 3D printing technology can synergistically optimize the mechanical properties and thermal insulation performance of porous ceramics according to application requirements [70]. For example, by changing key parameters such as printing path, layer thickness, and filling pattern, the precise regulation of porosity and pore size distribution of porous ceramics can be achieved, thereby significantly improving their thermal insulation capacity [71]; by embedding complex channels in porous ceramics and using cleverly designed channel geometries, heat flow can be effectively guided or blocked, avoiding the so-called “thermal bridge” phenomenon, and significantly improving the overall thermal insulation efficiency of porous ceramics. In addition, 3D printing can also provide possibilities for the preparation of multi-material or functionally graded ceramics, realizing advanced functions such as local thermal gradient regulation or mechanical enhancement within a single component [72].

The International Organization for Standardization (ISO) classifies 3D printing technologies into seven categories: Binder Jetting (BJ), Directed Energy Deposition (DED), Material Extrusion (ME), Material Jetting (MJ), Powder Bed Fusion (PBF), Sheet Lamination (SHL), and Vat Photopolymerization (VPP) [73]. Currently, the 3D printing technologies suitable for porous ceramic forming mainly include Binder Jetting [74,75,76,77,78], Material Jetting [79,80], Material Extrusion [81,82,83,84], and Vat Photopolymerization [85,86,87,88,89].

### 3.1. Binder Jetting

**Figure 6:**
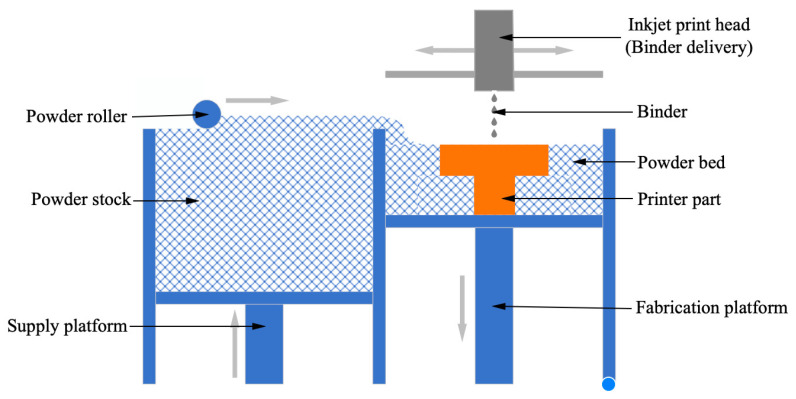
The principle of 3DP process.

Binder Jetting technology completes printing by selectively jetting and depositing liquid binders to bond powder materials. The representative process is Three-Dimensional Printing (3DP). 3DP was first proposed by Emanual Sachs from the Massachusetts Institute of Technology [90]. As shown in Figure 6, during forming, the 3D model of the part to be printed is input into slicing software to generate corresponding slice files according to the layer height. For single-layer printing, the powder-feeding cylinder platform rises, the forming cylinder platform lowers, and the powder-spreading roller spreads the powder from the powder-feeding cylinder to the forming cylinder. The nozzle above the forming cylinder orderly jets organic binder solution onto specific areas of the powder bed surface according to each layer of the model slice under computer control. The powder on the powder bed surface is bonded into a continuous solid after being infiltrated and wrapped by the binder. After the completion of single-layer printing, the single-layer printing is repeated until the model is fully formed. The printed green part is degreased to remove organic binders and other organic additives, and then sintered to obtain dense ceramic parts with good mechanical properties. No additional printing support is required during the 3DP printing process, as the powder in the forming cylinder can serve as model support during printing and be easily removed after printing.

Binder Jetting technology was initially designed to expand the range of materials that can be used by other 3D printing technologies (such as Selective Laser Sintering and Laminated Object Manufacturing), involving various powder materials such as ceramics [90], metals [91], plastics [92], and their composites [93]. With high flexibility in material selection and structural design, as well as the absence of the need for additional support structures during forming, Binder Jetting has been increasingly widely used in aerospace, biomedicine, and other fields.

**Figure 7:**
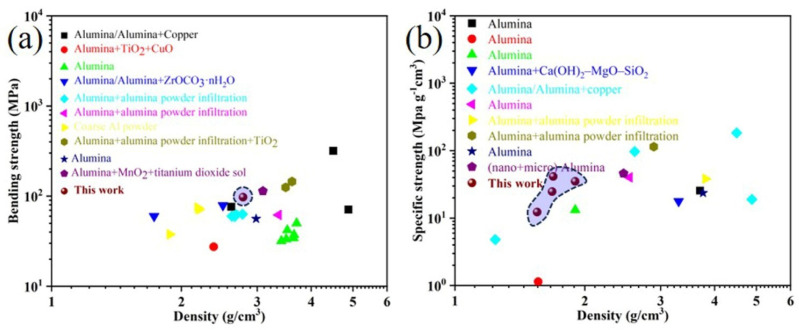
Comparison of properties of porous ceramics formed by BJ combined with multiphase infiltration process: (**a**) Bending strength, (**b**) Specific strength [94].

Wu et al. [94] combined Binder Jetting with a multiphase infiltration process. By filling fine powder and POSS (polyhedral oligomeric silsesquioxane) precursor during vacuum infiltration, toughening phases such as mullite were in situ generated after sintering, which effectively reduced the pore defects and significantly improved the strength of BJ-formed alumina ceramics. The porous ceramics prepared by this method showed significant improvements in both mechanical properties and thermal insulation performance. As shown in Figure 7, compared with other studies, the porous ceramics prepared in this study had a lower density at the same specific strength. Huang et al. [95] regulated the microstructure and mechanical properties of porous alumina ceramics formed by Binder Jetting by adding zirconium carbonate (ZBC) to the raw material powder. The experimental results showed that the zirconia particles produced by ZBC decomposition could fill the gaps between alumina particles and inhibit grain growth, thereby improving the strength of the sintered body while ensuring high porosity. The porous alumina ceramics formed by Binder Jetting with 6 wt% ZBC content achieved the optimal performance.

However, due to factors such as random agglomeration during forming, large friction between powders, and lack of external compaction, Binder Jetting products usually have problems such as poor forming quality and low mechanical properties [96], which greatly restrict their further application in the field of porous ceramics.

Notably, the inherent support-free forming characteristic and wide material compatibility of this technology make it uniquely advantageous for the low-cost, scalable fabrication of porous ceramic components with complex interconnected pore networks and tailorable porosity.

### 3.2. Material Extrusion

Material Extrusion technology converts ceramic raw materials into a flowable state by adding solvents or physical heating, and then extrudes them from a nozzle using external force to form the required parts through layer-by-layer stacking. The extrusion process of Material Extrusion is relatively simple, requiring low equipment and material costs, and has high cost-effectiveness. It also has a wide range of materials, including plastics, metals, ceramics, and concrete. However, it also has the problem of low forming quality, often with low strength and precision. According to the different printing methods and properties of printing materials, Material Extrusion can currently be divided into Fused Deposition Modeling (FDM) and Direct Ink Writing (DIW).

**Figure 8:**
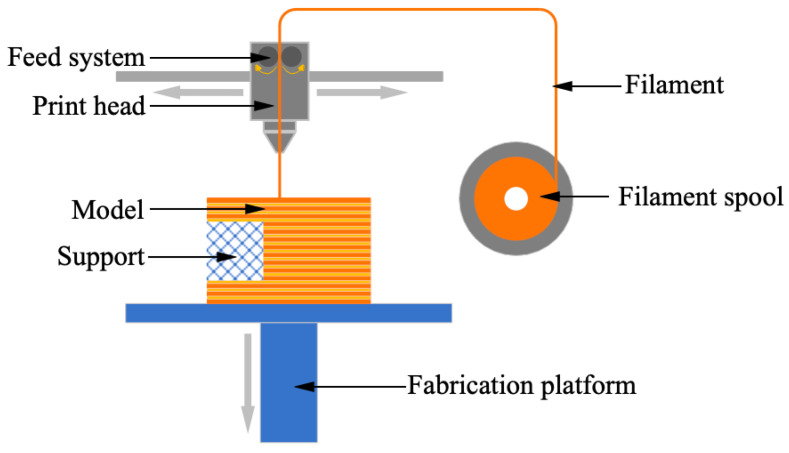
The principle of FDM process.

The principle of FDM is shown in Figure 8. A filament feeding mechanism feeds solid material filaments into a nozzle, which are heated to a molten state and then extruded and solidified along a predetermined path. After each layer is extruded, the micro-motion worktable moves for the next layer of printing until the part is formed. Since this technology was developed by Crump in the 1990s and commercialized by Stratasys [97], it has mostly been used to print thermoplastic polymer filaments with low melting points or some metal filaments with slightly higher melting points. Since ceramics themselves cannot be processed into filaments required for FDM, composite filaments formed by mixing a certain proportion of ceramic particles with thermoplastic binders are often used to achieve FDM forming.

Tosto et al. prepared ceramic parts by FDM using ceramic–polymer composite filaments containing 52 Vol% alumina particles. The prepared samples had an average density of 3.80 g·cm^−3^ (theoretical density of 3.94–3.95 g·cm^−3^, accounting for 96.5% of the theoretical density), with a tensile strength of 232.6 ± 12.3 MPa and a Vickers hardness of 21 ± 0.7 GPa, which were lower than those obtained by traditional processing [98]. De Toro et al. [99] experimentally proved that layer thickness plays an extremely important role in the flexural and impact properties of FDM-formed samples, while the influence of nozzle diameter on the samples is relatively small. To improve the flexural properties of the samples, reducing the layer thickness to enhance the bonding strength between layers can be considered, while increasing the layer thickness can achieve better impact properties to a certain extent, which needs to be balanced according to actual application conditions. Baich et al. [100] studied the correlation between filling structure and forming performance in their work. Different filling structures can improve some mechanical properties of the formed samples. Therefore, during the design stage, the filling structure can be optimized according to the application scenario of the part to give full play to the performance of the part in specific scenarios.

Although FDM has a simple forming process and low equipment and material costs, problems such as cavities and pores between layers [101], anisotropy caused by the forming principle [80], and step effect in the Z direction [102] limit its application and development in porous ceramic forming in terms of surface roughness, dimensional accuracy, and mechanical properties.

**Figure 9:**
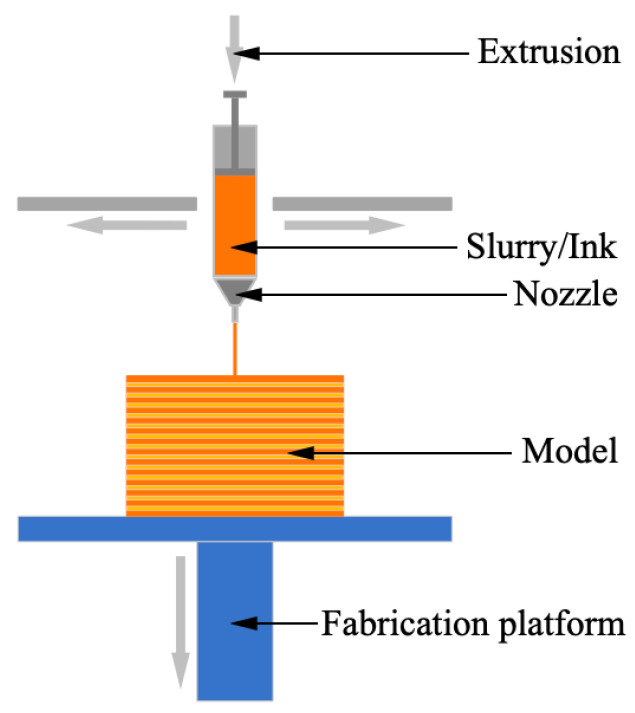
The principle of DIW process.

Compared with FDM, Direct Ink Writing (DIW) has more advantages in forming porous ceramics due to the complex and cumbersome filament preparation process [40]. DIW technology was first proposed and patented by CESARANO et al. in 1997 [103]. Different from FDM, which melts and extrudes filaments, DIW directly extrudes and forms ceramic slurries mixed with ceramic powder and binders. The forming principle of this technology is shown in Figure 9. Ceramic slurries for DIW usually have a high solid content and high viscosity to ensure that they have a certain strength after extrusion forming and can maintain their shape during the deposition of the next layer. This characteristic also makes DIW more advantageous than other 3D printing technologies in forming overhanging structures, i.e., no support structure is required.

On the other hand, the deposition process of DIW is mainly carried out at room temperature, so the formed parts are not affected by thermal stress and residual stress [104] and have good mechanical properties. Yu et al. conducted a study on DIW forming of partially stabilized zirconia ceramics (3 mol% Y_2_O_3_) with high solid content (60%) slurry. After sintering, the relative density of the formed parts reached 98.1%, and the flexural strength, fracture toughness, compressive strength, and Vickers hardness were 488.96 ± 79.84 MPa, 2.63 ± 0.2 MPa·m^1/2^, 1.56 GPa, and 11.52 ± 0.57 GPa, respectively, which were higher than the mechanical properties of parts formed by Binder Jetting reported in the literature [105].

**Figure 10:**
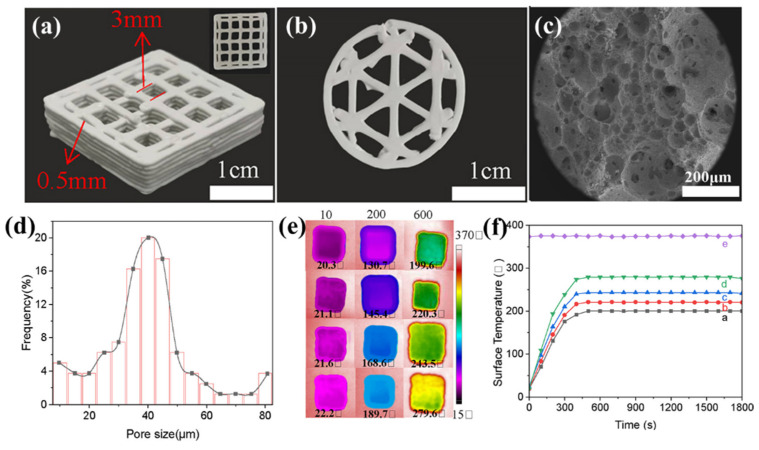
Fabrication of alumina ceramics by DIW and their thermal insulation performance: (**a**,**b**) 3D printed gel foams with different lattice shapes. (**c**) SEM image of filaments. (**d**) Micron-scale pore size distribution in DIW-formed alumina foams. (**e**) Infrared thermal imaging during thermal insulation test with different temperatures (°C). (**f**) Surface temperature (°C) of foam ceramics with different average sizes [106].

There have been many applications of DIW in forming porous ceramics. Yang et al. [106] prepared Al_2_O_3_ ceramics with hierarchical pore structures as shown in Figure 10a–c by DIW. By adding different contents of Ammonium Oleate to the colloidal gel, the micropore size could be reduced from 137.4 μm to 41.5 μm, thereby obtaining adjustable thermal insulation performance. When the average micropore size was 41.5 μm, the DIW-formed foam ceramics had the best thermal protection capability: on a hot stage at 380 °C, the surface temperature was only 220.3 °C (significantly lower than 279.6 °C without thermal insulation), as shown in Figure 10e,f.

Miao et al. [107] used DIW to print a mixed slurry containing carbon fibers, SiO_2_, and a Al_2_O_3_ precursor, and grew SiC nanowires in situ at a high temperature to obtain a mullite-reinforced SiC aerogel. This material could maintain a Young’s modulus of 24.4 MPa and a compressive strength of 1.65 MPa at 90% porosity, with a thermal conductivity as low as 0.021 W·m^−1^·K^−1^.

**Figure 11:**
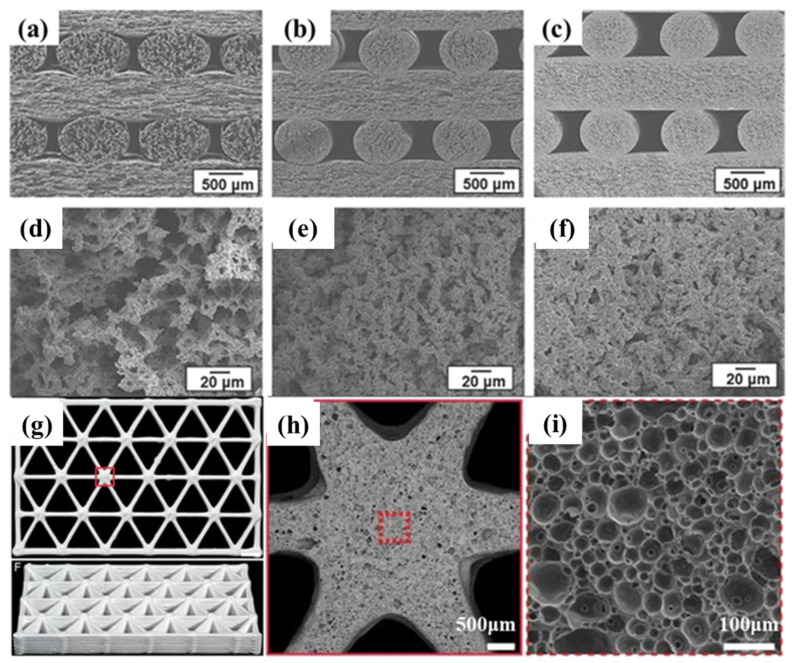
Fabrication of multi-layer porous ceramics by DIW combined with traditional preparation techniques: (**a**–**f**) FE-SEM images of porous ceramics formed by DIW combined with freeze-casting at different alumina contents: (**a**,**d**) 15 vol%, (**b**,**e**) 20 vol%, (**c**,**f**) 25 vol% [108]. (**g**–**i**) SEM images of porous ceramics formed by DIW combined with direct foaming [109].

In addition, many studies have reported the combination of DIW with the aforementioned traditional preparation technologies to achieve the preparation of hierarchical porous ceramics. Moon et al. [108] successfully formed hierarchical porous alumina ceramic scaffolds with 3D interconnected macropores and aligned micropores as shown in Figure 11a–e by combining DIW with freeze-casting technology. The macropores were interconnected with a pore size range of approximately 150 µm to 600 µm, and the size of the micropores could be adjusted according to the initial alumina content. When the alumina content increased to 25 vol%, the micropore size could reach 0.3–4 µm. Muth et al. [109] introduced direct foaming technology into DIW, generating stable bubbles in colloidal suspensions to print porous ceramic structures with periodic unit structures such as triangular honeycombs as shown in Figure 11g,h, realizing the dual design of microscale closed pore structure and macroscale geometric shape. By adjusting the foaming content and unit topology, hierarchical porous ceramics can be obtained at a low density, with high specific stiffness and controllable elastic modulus spanning more than an order of magnitude.

Since DIW uses ceramic slurries with a high solid content, to ensure the printability of the slurry and prevent nozzle clogging, the nozzle size must be at least 15 times the maximum particle size [110], making a large-diameter nozzle indispensable. However, a large-diameter nozzle will bring problems such as obvious step effect and low surface quality to the formed parts. Although some surface treatment methods can effectively improve the surface quality of DIW-formed parts, there are still some issues to be solved in terms of overall forming efficiency and geometric accuracy.

For porous microstructure production, this technology excels in the support-free fabrication of overhanging porous frameworks and hierarchical porous structures, enabling the integrated customization of macro-scale porous geometry and micro-scale pore morphology at room temperature without thermal stress damage.

### 3.3. Vat Photopolymerization

**Figure 12:**
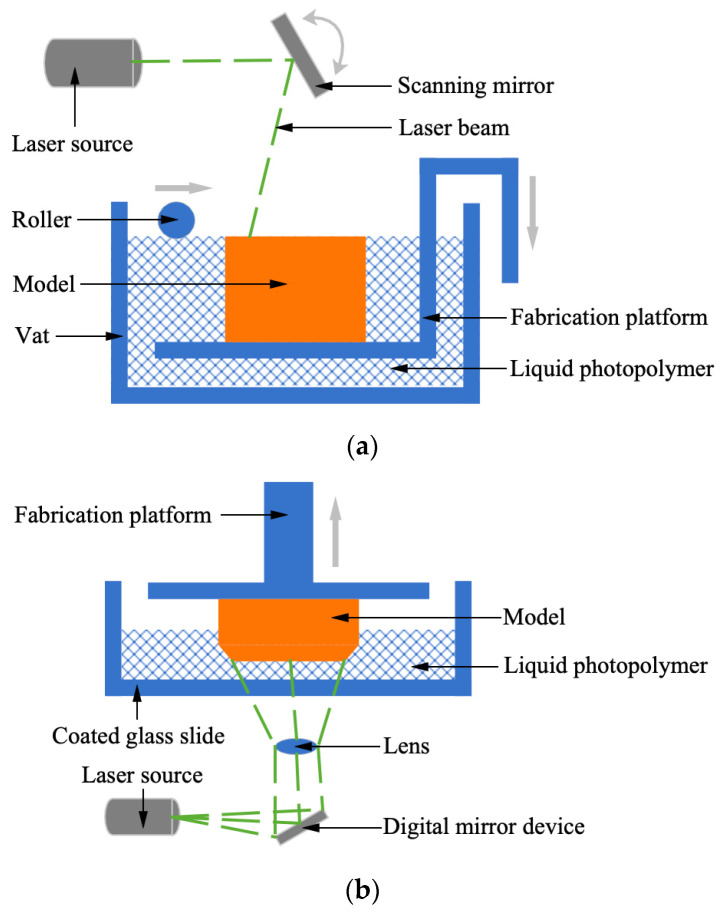
The principle of VPP process: (**a**) SLA, (**b**) DLP.

As one of the current mainstream 3D printing technologies, Vat Photopolymerization is widely used in metamaterials, functional devices, biomedicine, and other fields. Currently, Vat Photopolymerization is mainly divided into SLA, Digital Light Processing (DLP), and Two Photon Polymerization (TPP) [111]. The specific forming principles are shown in Figure 12. Among them, TPP, as a micro-nano 3D printing technology, is often used to manufacture high-performance parts with nanoscale features, and its typical application is currently in the field of microelectronics. Considering its forming feature size, TPP will not be elaborated on in this study.

During the ceramic-forming process using SLA and DLP, ceramic powder is often mixed with photosensitive resin to form a ceramic slurry system. After irradiation with light of a specific wavelength, the photosensitive resin polymerizes and crosslinks to form a network structure that uniformly wraps and disperses the ceramic powder, realizing the macro curing of the ceramic slurry. The formed part is then subjected to high-temperature degreasing and sintering to further densify it, forming the final ceramic sample.

SLA technology was first proposed by Hull in 1986 [112], and later successfully commercialized by 3D Systems with corresponding equipment launched. As shown in Figure 12a, its forming principle involves scanning and curing the surface of the aforementioned ceramic photosensitive slurry with a light beam of a specific wavelength, leveling the slurry surface with a scraper, etc., and stacking layer by layer to form a ceramic green part, which is then degreased and sintered to densify into the final ceramic part. The light beam spot used in this technology is only 60–140 μm, so the surface quality of the formed parts is generally high.

DLP technology was first proposed by Nakamoto et al. from Japan [113]. Essentially, it is a mask-based SLA technology that cures the entire layer of the printed shape onto the surface of the aforementioned ceramic photosensitive slurry through a mask after layering. Initially, DLP was realized through solid masks and liquid crystal display (LCD) dynamic masks. With the emergence of Digital Mirror Device (DMD), it has gradually replaced LCD as the new generation of DLP mask technology [114]. The forming principle of DLP based on DMD is shown in Figure 12b. Compared with LCD, DMD has more advantages in fill factor, reflectivity, and other aspects. DLP technology can not only achieve micron-scale forming resolution but also has a shorter forming time and higher forming efficiency compared with the point-line-surface scanning and curing strategy of SLA.

**Figure 13:**
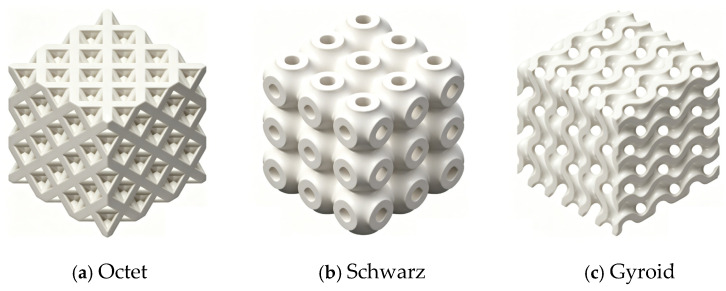
DLP fabrication of porous ceramics with different TPMS structures.

Compared with the aforementioned Material Extrusion or Binder Jetting technologies, Vat Photopolymerization can achieve higher precision and smoother surfaces. In recent years, significant progress has been made in the preparation of porous ceramics. Researchers have continuously optimized its system and explored its advantages in mechanical properties and thermal insulation performance. For example, in terms of thermal insulation performance, Yang et al. [115] successfully prepared 3Y-TZP porous ceramics with Triply Periodic Minimal Surface (TPMS) structures using DLP. By regulating the TPMS pore structure and porosity, as shown in Figure 13, a balance can be achieved between the mechanical properties and thermal insulation performance of porous ceramics. While effectively reducing the thermal conductivity of porous ceramics, high mechanical properties are maintained. The research data shows that under the diamond-type TPMS structure with 67% porosity, the porous ceramics formed by DLP can achieve a thermal conductivity of 0.93 ± 0.035 W·m^−1^·K^−1^ and a compressive strength of 166.5 ± 27.3 MPa.

Chao et al. [116] developed a porous ceramic based on mullite fibers using DLP, which has a complex geometric structure and excellent high-temperature thermal insulation performance. By using photosensitive hydroxyl silicone oil (HPMS-KH570) as the resin matrix, the uniform dispersion and structural stability of mullite fibers are achieved. When the aspect ratio of the fibers is 45 and the volume content is 6.67%, the material exhibits the best performance, with a density of 0.47 g·cm^−3^ and a thermal conductivity of 0.11 W·m^−1^·K^−1^. In addition, the material maintains stable thermal insulation performance in the high-temperature range of 1000 to 1400 °C, and no significant increase in thermal conductivity occurs even at 1200 °C.

In terms of mechanical properties, the research by Mao et al. [117] provides a new method for preparing high-performance textured porous Si_3_N_4_ ceramic devices using Vat Photopolymerization technology. By combining Vat Photopolymerization with the seed crystal method, porous silicon nitride ceramics with a porosity of 28.41% and significantly improved mechanical properties can be prepared, with a maximum flexural strength of 272 ± 17 MPa and a fracture toughness of 5.65 ± 0.2 MPa·m^1/2^. Kong et al. [118] proposed to impregnate and modify alumina porous ceramics formed by Vat Photopolymerization and sintering with permeable polymers to obtain a bionic structure similar to the hammer-like dactyl club of mantis shrimp. The experimental results show that the impregnated porous ceramics not only have significantly improved compressive strength but also exhibit non-brittle fracture characteristics and excellent energy absorption effects.

Although Vat Photopolymerization has high forming precision in the preparation of porous ceramics, due to the particularity of its materials, the decomposition of a large amount of photosensitive resin in the green part during the degreasing and sintering process results in a high shrinkage rate of the sample, which is prone to problems such as cracks, pore collapse, and even cracking [119], especially in porous structures. In addition, the equipment for Vat Photopolymerization has certain limitations on the forming size and complexity, especially in the construction of large-scale and multi-layer pore structures, which are prone to problems such as incomplete curing and edge distortion.

With its ultra-high micron-scale forming resolution and excellent surface quality, this technology offers unparalleled advantages for the precise fabrication of complex periodic porous microstructures (such as TPMS architectures) and fine pore walls, achieving the synergistic optimization of thermal insulation and mechanical properties of porous ceramics.

### 3.4. Material Jetting

**Figure 14:**
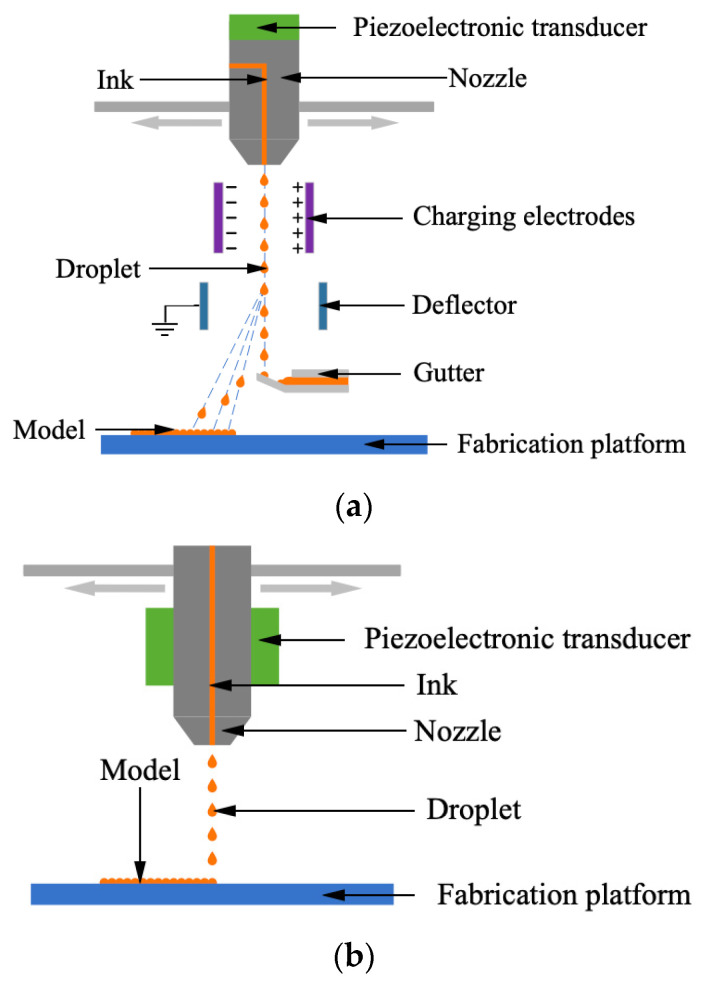
The principle of MJ process: (**a**) CIJ, (**b**) DOD.

Material Jetting is usually also known as inkjet printing, derived from inkjet printing used for printing plane text and images in daily life. It jets ink containing material components onto the forming platform using a method similar to inkjet printers, and then builds the target 3D model layer by layer using curing methods such as photopolymerization or thermal curing. According to different working methods, Material Jetting can be divided into Continuous Ink-jet Printing (CIJ) and Drop-on-Demand Ink-jet Printing (DOD) as shown in Figure 14.

In CIJ, piezoelectric elements provide constant pressure to the nozzle to make ink eject from the nozzle at a high speed in the form of droplets. Under the action of the charging electrode, the droplets are charged with corresponding charges. The flight path of the droplets can be controlled by controlling the deflection electric field. During the movement of the nozzle, when the worktable under the nozzle needs to be printed, the deflection electric field is applied to control the droplets to fly to the worktable; if no printing is needed, the deflection electric field is stopped to make the droplets fly into the recovery device. The droplets flying to the worktable are bonded and cured to form the printed model. The forming principle of CIJ is shown in Figure 14a.

Compared with CIJ, DOD nozzles usually have more nozzles, which can produce smaller and more precise droplets, thereby achieving higher-quality printing, as shown in Figure 14b. At the same time, since DOD only needs to jet ink after reaching a specific position, the printing process does not need to cover the entire printing page, which has more advantages in printing efficiency and ink consumption. Therefore, most current studies on Material Jetting technology adopt DOD.

At present, most studies on Material Jetting focus on process exploration. For example, Willems et al. [79] systematically analyzed the microstructure and mechanical properties of formed samples around the process of preparing 3Y-TZP zirconia ceramics by Material Jetting. They found that although the zirconia ceramics formed by Material Jetting have high density and mechanical properties, the flexural strength of the samples shows a strong dependence on the building direction due to the delamination defects at the interlayer interface and the high surface roughness of the samples in the 45° and 90° building directions. Optimizing the ink design by adjusting the ink composition and solid content, and adjusting the printing parameters during the droplet deposition process to improve the bonding between green layers during forming are the keys to improving the performance of MJ-formed zirconia ceramics.

Fayazfar et al. [120] developed a new DOD forming scheme for partially stabilized zirconia (5Y-TPZ) containing 8 wt% Y_2_O_3_ by regulating the slurry composition and rheological properties, optimizing the printing path and sintering method. This scheme can directly print zirconia slurry with high viscosity and a solid content of up to 72 wt%. The formed zirconia ceramic samples have a relative density of 99.5%, no obvious abnormal grain growth, low porosity with uniform distribution, a hardness of 1516 HV, and a fracture toughness of about 5.62 MPa·m^1/2^, which meet the mechanical requirements for high-performance zirconia-based ceramics such as dental restoration.

In addition, a few studies have reported that NanoParticle Jetting (NPJ), a branch of Material Jetting, has made significant progress in ceramic forming. This technology jets liquid “ink” dispersed with nanoparticles and rapidly evaporates the ink solvent at a high temperature, enabling more efficient and high-precision forming effects [80,121,122]. Mummareddy et al. [123] prepared zirconia ceramic solid parts and lattice structures using NPJ. The study found that different sintering processes and printing directions significantly affect the material properties. The 0° printing direction shows better compressive strength (average about 1565 MPa) and flexural strength (about 430 MPa); at the same time, Hot Isostatic Pressing (HIP) treatment can effectively repair internal crack defects.

**Figure 15:**
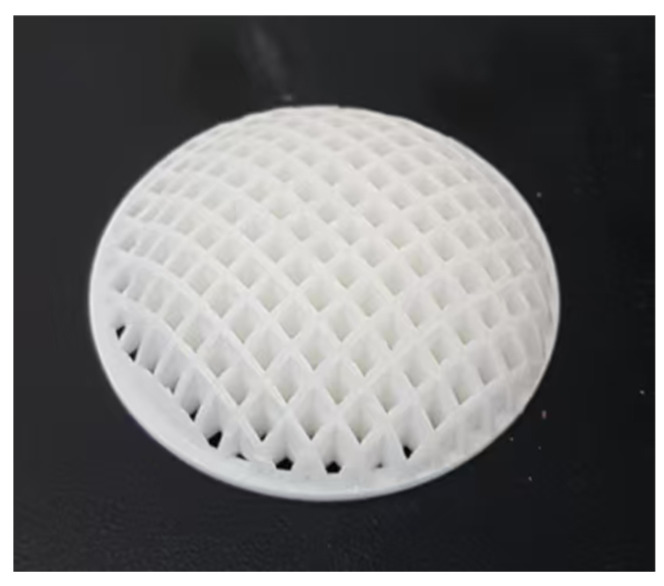
Fabrication of 5G radomes by NPJ.

Gofman et al. [124] successfully prepared porous zirconia ceramic membranes with smooth surfaces using NPJ technology, with an average roughness of 0.33 μm, and achieved the forming of micron-scale pores. In our lab, NPJ printing was used to produce 5G radomes, which can achieve gradual changes in dielectric constant. Benefiting from this adjustable dielectric gradient, the electromagnetic property of 5G antenna components gets effectively optimized, as shown in Figure 15.

Table 1 and Table 2 summarize the typical precision, dimensions, advantages, and disadvantages of the four main 3D printing technologies for porous ceramic materials. Compared with the other three technologies, Material Jetting has the advantages of good forming precision, large forming size, fast forming speed, and high material utilization rate. However, during its preparation process, it involves multiple physical and chemical processes such as ink jetting, droplet deposition and curing, degreasing, and sintering, and the final forming performance is greatly affected by the process.

Taking the ink jetting process as an example, due to the high density and easy sedimentation of ceramic particles in high-solid-content ink, the ink is prone to problems such as nozzle clogging and uneven deposition during the jetting process, which is more significant when preparing high-porosity structures. In addition, porous ceramic structures have higher requirements for jetting precision and interlayer bonding. Microdroplet deviation, splashing, or insufficient fusion during the jetting process may lead to pore structure distortion or even functional failure. The performance of the green part formed by Material Jetting largely depends on the performance of the jetted droplets. Even with the same nozzle, the performance and stability of the droplets are not only affected by the rheological properties of the ink such as dispersibility, stability, viscosity, and surface tension [102,129,130,131] but also by the driving voltage waveform structure and waveform parameters of the piezoelectric actuator on the piezoelectric nozzle [132,133,134,135,136,137,138,139].

Therefore, in-depth research on the key influencing factors in the Material Jetting process, especially the systematic exploration of the construction of stable jetting mechanisms for high-solid-content ceramic inks and the control of complex structure forming processes, is of great theoretical significance and practical value for improving the preparation quality of porous ceramics and expanding their engineering applications in fields such as thermal insulation and structure–function integration. For porous ceramic manufacturing, this technology’s high droplet-level deposition precision, excellent forming uniformity, and high material utilization rate make it particularly suitable for the high-precision batch fabrication of porous ceramic membranes and functional porous components with micron-scale controllable pore structures.

## 4. Structure–Property Optimization of 3D-Printed Porous Ceramics

3D printing technology provides porous ceramics with degrees of freedom difficult to achieve by traditional manufacturing methods in constructing complex geometric structures, designing high porosity, and integrating functions. It not only has high forming precision and repeatability but also endows porous ceramics with more possibilities for structural topology optimization and performance collaborative design. As a key link to improve the thermal and mechanical properties of 3D-printed porous ceramics, structure optimization is gradually becoming a research hotspot in this field. 3D-printed porous ceramics generally have regular structures, so their optimization process often involves several key steps: first, the 3D printing model needs to be carefully designed, and parameters such as pore size, shape, distribution, and interconnectivity should be reasonably set to achieve the expected performance; then, appropriate printing materials and printing processes are selected to ensure effective control of the pores of porous ceramics during printing; after printing and forming, the pore structure is further optimized through post-processing processes such as sintering and surface treatment; finally, comprehensive characterization methods including microscope observation and thermal and mechanical performance tests are used to verify whether the performance of the optimized porous ceramics meets the expectations. In this process, computational modeling and simulation optimization are indispensable tools, playing a crucial role in improving the thermal and mechanical properties of 3D-printed porous ceramics.

The process–structure–property relationship of 3D-printed porous ceramics is intrinsically correlated: 3D printing process parameters (e.g., binder dosage in Binder Jetting, ink viscosity in Material Extrusion, curing intensity in Vat Photopolymerization, and droplet stability in Material Jetting) directly determine the forming quality and pore characteristics (porosity, pore size, pore connectivity, and TPMS topological structure). In turn, these structural features govern the macroscopic thermal insulation and mechanical properties. Rational regulation of printing processes enables precise tailoring of pore structures, which further breaks the trade-off between thermal insulation and mechanical strength. Computational modeling quantitatively links process variables, structural parameters, and final properties, providing a reliable basis for the targeted design and preparation of high-performance porous ceramics.

Sun et al. successfully prepared Si/SiC ceramic components with gradient pore structures using 3D printing technology and systematically studied the influence of pore gradient changes on mechanical properties. In the study, various Gyroid-type TPMS structures with different gradient transition rates and gradient spans were designed. The regulation mechanism of different structural parameters on the failure mode, stress distribution, and overall performance was revealed through quasi-static compression tests and finite element simulations. In addition, a performance prediction method combining laminated material theory and the Gibson–Ashby model was constructed to realize the quantitative prediction of the mechanical properties of gradient structures. Research results confirmed that reasonable design of gradient structures can significantly improve the strength, stiffness, and reliability of gradient porous ceramic components, providing a theoretical basis and manufacturing path for their application in the direction of structure–function integration [140]. DiReda et al. studied the thermal–structural performance of five TPMS structures in ceramic porous media burners. Through thermal–mechanical coupling simulation and DLP printing experiments, they found that different TPMS structures have significant differences in stress distribution and crack resistance. Among them, the Diamond and I-WP structures have better thermal stability and mechanical reliability. The accuracy of the simulation in predicting high-risk crack areas was verified by X-ray CT [141].

Yang et al. used finite element simulation to compare and analyze the thermal insulation and compression properties of porous 3Y-TZP ceramics with different TPMS structures (Diamond, Gyroid, Schwartz P). The simulation results showed that the Diamond structure has the lowest thermal conductivity and the highest mechanical strength at the same porosity. In addition, the study further explored the influence of porosity and the number of periods on the performance of porous ceramics, providing simulation support and structural design basis for the collaborative optimization of thermal and mechanical properties of porous ceramics [115]. Liang et al. analyzed the flow field and temperature field of three different structures (Tetrakaidecahedron, Octahedron, Cubic) of SiC porous ceramics in a combustion environment through simulation. The results revealed the influence of porous structures on airflow disturbance, pressure drop, and heat exchange efficiency. Among them, the Tetrakaidecahedron configuration showed the best heat transfer performance and combustion stability [142]. Zhou et al. studied the static and dynamic mechanical properties of three ceramic lattice structures at different relative densities using finite element simulation combined with experiments. A brittle fracture model and element deletion technology were introduced in the simulation to accurately predict the structural stress distribution and failure behavior. The results showed that the TOH structure has better stress uniformity and load-bearing performance in the vertical loading direction, and the simulation results are highly consistent with the experiments, providing a theoretical basis for the structural optimization of porous ceramics under complex loads [143].

With the help of computational modeling and simulation optimization methods, in sharp contrast to the empirical, trial-and-error structural design mode relied on in traditional porous ceramic preparation (which can only conduct superficial and qualitative judgment on the structure–property relationship and is unable to accurately analyze the internal stress and heat transfer law of materials), we can conduct in-depth and quantitative simulation analysis on the stress distribution, deformation response and heat conduction behavior of porous ceramic materials under different pore structure parameters (e.g., porosity, pore size, topological structure and gradient distribution). By virtue of such precise digital simulation, we can effectively reveal the intrinsic influence mechanism of various structural parameters on the thermal insulation and mechanical properties of porous ceramics that cannot be identified by traditional experimental methods alone, accurately screen out the optimal pore structure combination matching the service requirements under specific working conditions, and thus realize the scientific and targeted balance optimization between the thermal insulation performance and load-bearing capacity of porous ceramics—breaking the long-standing dilemma in traditional preparation where performance optimization can only be achieved at the expense of one another due to the lack of accurate structural regulation means. Taking Material Jetting technology as the core manufacturing path in this study, we further address the shortcomings of other mainstream 3D printing technologies for porous ceramics (e.g., low mechanical strength of Binder Jetting, poor forming precision of Material Extrusion, high shrinkage and difficult degreasing of Vat Photopolymerization) in the integration of structural design and manufacturing process. We construct a complete and systematic technology chain for MJ-based preparation of porous ceramic components covering theoretical modeling, equipment optimization, process parameter realization and structure–property collaborative design. This integrated technology chain makes up for the fragmentation of research on “design-manufacturing-performance” of 3D-printed porous ceramics in existing studies, and provides important theoretical support and technical guidance for the intelligent digital design and high-quality, high-precision preparation of high-performance complex structure ceramics for high-value application scenarios such as aerospace and new energy.

## 5. Conclusions

This review systematically summarizes the research progress of 3D-printed porous ceramics in preparation technology, structure–property optimization and performance regulation, clarifies the scientific contributions of this work, and explicitly addresses the key gaps in the current literature, including lack of standardization, limited experimental/industrial validation and methodological inconsistencies, providing a targeted reference for the field’s research and engineering application.

Traditional porous ceramic preparation methods are limited by uncontrollable pore structures and unstable mechanical properties, while 3D printing enables precise regulation of pore microstructures and fabrication of complex structures. This review conducts a comparative analysis of four mainstream 3D printing technologies (Binder Jetting, Material Extrusion, Vat Photopolymerization, Material Jetting) for porous ceramics, elaborating their forming mechanisms, performance pros and cons, and typical applications, and quantifying their forming accuracy and applicable dimensions via standardized tables. This work addresses the methodological inconsistencies in current research (disparate process parameters and evaluation criteria leading to poor result comparability) and fills the gap of insufficient standardization in technical comparison, offering a clear basis for rational selection of preparation technologies.

As the core scientific contribution, this review systematically collates the structure–property optimization strategies of 3D-printed porous ceramics, focusing on the application of Triply Periodic Minimal Surface (TPMS) design and computational modeling/simulation in balancing thermal insulation and mechanical properties. It also summarizes the fabrication of hierarchical porous ceramics by combining 3D printing with traditional methods, filling the gap of lacking systematic combing of optimization methods, and providing a practical design reference for the synergistic regulation of porous ceramics’ key properties.

This review further identifies the key unresolved issues in the current field: no unified industry standards for ceramic ink formulation, process parameters and performance characterization, resulting in poor research reproducibility; most studies are limited to laboratory-scale small samples, with insufficient experimental and industrial validation for large-sized and complex structural components; technical bottlenecks such as poor high-solid-content ink stability, nozzle clogging and high sintering shrinkage still restrict the large-scale production of 3D-printed porous ceramics.

In view of the above gaps and challenges, future research priorities include: optimizing ceramic ink formulations to improve printability and stability; developing advanced degreasing and sintering technologies to reduce shrinkage and cracking of porous structures; establishing unified industry standards to solve the problems of non-standardization and methodological inconsistency; strengthening the experimental verification of large-sized components and promoting industrial application in aerospace, new energy and other high-value fields; and integrating artificial intelligence to realize intelligent optimization of structural design and printing processes.

In conclusion, this review clarifies its scientific value in filling the key research gaps of the field, and constructs a technical framework from preparation technology selection to structure–property optimization. It provides a concise and systematic reference for the basic research and engineering transformation of 3D-printed porous ceramics, and is expected to promote their wider application in energy conservation, aerospace, biomedicine and other high-tech fields.

## Figures and Tables

**Table 1:** Accuracy and dimensions of 3D printing technologies for porous ceramics.

3D Technologies	Accuracy	Dimension (mm)
FDM [80]	>100 µm	≈350 × 250 × 200
DIW [125]	>100 µm	≈150 × 150 × 140
SLA [126]	10–100 µm	≈320 × 3200 × 200
DLP [127]	5–50 µm	≈180 × 180 × 100
BJ [128]	>100 µm	≈160 × 65 × 65
MJ [80]	≈20 µm	≈500 × 280 × 200

**Table 2:** Advantages and disadvantages of 3D printing technologies for porous ceramics.

3D Technologies	Advantages	Disadvantages
BJ	No support required Small size limitation Fast forming speed Wide range of usable materials	Toxic binder Poor forming precision Low mechanical properties of formed parts Easy nozzle clogging
ME	Low equipment cost Small size limitation Wide range of usable materials	Support required Poor forming precision Easy nozzle clogging Slow forming speed
VPP	High surface quality of formed parts High forming precision Fast forming speed Wide range of usable materials	Toxic materials Low material utilization rate Difficult degreasing
MJ	High forming precision High surface quality of formed parts Fast forming speed High material utilization rate	Easy nozzle clogging High equipment cost Difficult ink storage Support required

## Data Availability

No new data were created or analyzed in this study. Data sharing is not applicable to this article.
